# Bioapplications of Magnetic Nanowires: Barcodes, Biocomposites, Heaters

**DOI:** 10.1109/tmag.2022.3151608

**Published:** 2022-02-23

**Authors:** Mohammad Reza Zamani Kouhpanji, Yali Zhang, Joseph Um, Kartihik Srinivasan, Anirudh Sharma, Daniel Shore, Zhe Gao, Yicong Chen, Allison Harpel, Zohreh Nemati Porshokouh, Thomas E. Gage, Oana Dragos-Pinzaru, Ibro Tabakovic, P. B. Visscher, John Bischof, Jaime F. Modiano, Rhonda Franklin, Bethanie J. H. Stadler

**Affiliations:** 1 Department of Electrical and Computer Engineering, University of Minnesota, Minneapolis, MN 55455 USA; 2 Department of Chemical Engineering and Materials Science, University of Minnesota, Minneapolis, MN 55455 USA; 3 Department of Mechanical Engineering, University of Minnesota, Minneapolis, MN 55455 USA; 4 National Institute of Research and Development for Technical Physics, 700050 Iasi, Romania; 5 Department of Physics and Astronomy, The University of Alabama, Tuscaloosa, AL 35401 USA; 6 Department of Veterinary Clinical Sciences, College of Veterinary Medicine, University of Minnesota, Falcon Heights, MN 55108 USA; 7 Masonic Cancer Center, University of Minnesota, Minneapolis, MN 55455 USA

**Keywords:** Barcodes, biocomposites, first-order reversal curves (FORCs), magnetic nanowires (MNWs), nanowarmers, projection method

## Abstract

Magnetic nanowires (MNWs) can have their moments reversed via several mechanisms that are controlled using the composition, length, diameter, and density of nanowires in arrays as-synthesized or as individual nanoparticles in assays or gels. This tailoring of magnetic reversal leads to unique properties that can be used as a signature for reading out the type of MNW for applications as nano-barcodes. When synthesized inside track-etched polycarbonate membranes, the resulting MNW-embedded membranes can be used as biocompatible bandaids for detection without contact or optical sighting. When etched out of the growth template, free-floating MNWs are internalized by cells at 37 °C such that cells and/or exosomes can be collected and detected. In applications of cryopreservation, MNWs can be suspended in cryopreservation agents (CPAs) for injection into the blood vessels of tissues and organs as they are vitrified to –200 °C. Using an alternating magnetic field, the MNWs can then be nanowarmed rapidly to prevent crystallization and uniformly to prevent cracking of specimens, for example, as grafts or transplants. This invited paper is a review of recent progress in the specific bioapplications of MNWs to barcodes, biocomposites, and nanowarmers.

## I. Introduction

**Fig. 1.**
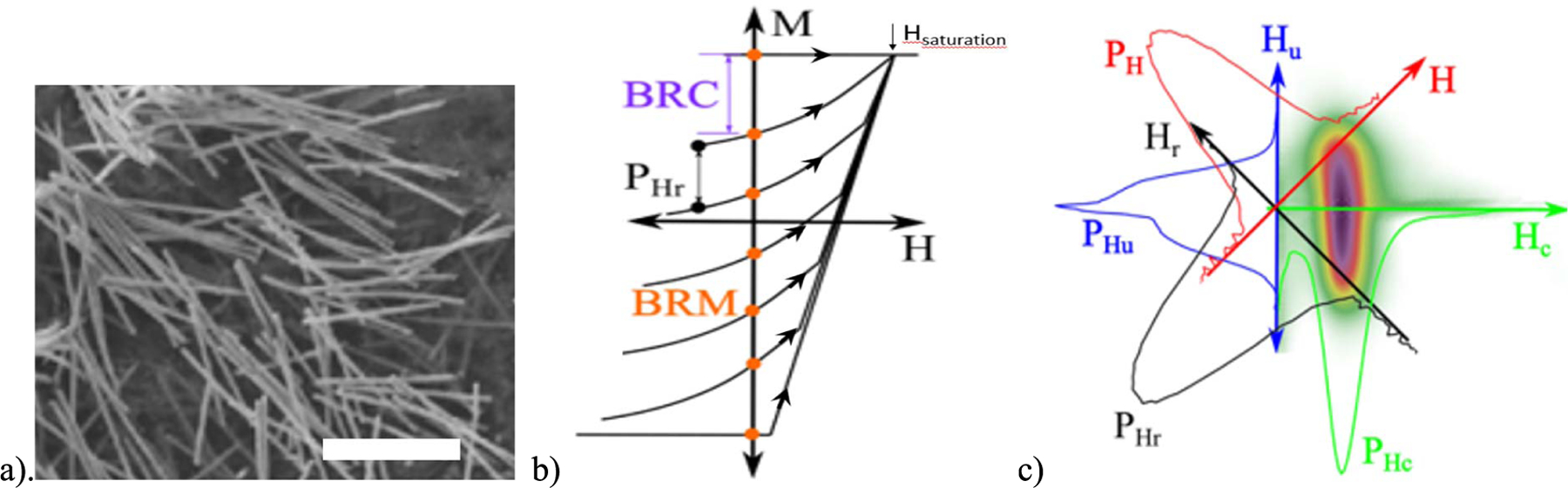
(a) Typical image of MNWs (scale bar = 1 *μ*m). Recall that a red blood cell is 6× larger than the length of these MNWs that are 100× larger than our smallest MNWs. (b) Schematic of FORC measurements (black lines showing the direction of field sweeps). (c) Typical FORC parameter (1) for MNW arrays plotted as a heatmap versus the coercivity (*H*_*c*_) and interaction fields (*H*_*u*_). Various parameters for distinguishing barcode MNWs are also shown and described in the text (*P*_Hr_, *P*_*H*_, *P*_Hu_, *P*_Hc_, BRM, and BRC) [32].

MAGNETIC nanowires (MNWs), see Fig. 1(a), are synthesized by electrochemical deposition into columnar nanopores, such as those found in track-etched polycarbonate (TEPC) or anodic aluminum oxide (AAO). Synthesis details can be found in recent reviews by Vazquez [1], [2]. Various shapes can be engineered, including modulated diameters and compositions by Escrig *et al.* [3], [4], core–shell geometries by Kosel [5], and 3-D networks by Piraux [6]. Applications include computing, logic or storage [7], MRI contrast [8], and the biolabels and nanowarmers presented in this article. Each of these applications benefits from a deep knowledge of the reversal mechanisms, which have been calculated by researchers, including Chubykalo–Fesenko [9] and observed by others such as Bloch-point domain walls by Fruchart [10] and layer reversal by Flatau [11].

As deposited, the nanoporous growth membranes inherently enable arrays of vertically aligned MNWs with large shape-induced magnetic anisotropies and interwire interactions that provide coding parameters for read-out via non-contact methods [12]. MNWs can also be released [Fig. 1(a)] from their growth membranes and subsequently incorporated into gels for 3-D printing and additive manufacturing [13]. Free-floating MNWs in suspensions or assays will be internalized by cells [14], [15] which then dispense exosomes [16], both of which can be subsequently collected with an external magnet [17] or read-out by similar methods as used for MNWs in membranes [12]. A very recent application of MNWs is nanowarming of cryopreserved tissues and organs, which enables the freezing of biological time [18]. Bioapplications of MNWs are wide-ranging and cannot be covered well in this article alone. The specific applications of barcodes, biocomposites, and nanowarmers will be discussed in the following, where it should be noted that MNWs are inside templates and/or coated with biomolecules.

## II. Barcodes

MNWs of Co, Fe, FeCo, and Ni have been synthesized in AAO and in nanoporous polycarbonate, the latter of which is biocompatible and has been used as an internal “bandaid” for organs [12]. Magnetization reversal of MNWs typically occurs by the motion of transverse or vortex domain walls. In small-diameter MNWs, the shape of the MNWs causes the magnetization to point either fully “up” or “down” unless the MNWs are segmented along their lengths, and the coercivity exhibits a strong inverse dependence on the MNW diameter [19]. With larger diameters (100–200 nm), vortices can wrap around the entire MNW lengths and are sometimes referred to as skyrmion lines because the center remains directionally “up” along the whole length [8]. This variance in reversal mechanisms enables intentional coding as the coercivity and hysteretic behavior are engineered for distinct signatures. Zamani *et al.* [20]–[22] recently explored a variety of measurement methods that can detect these signatures, all of which started with the measurement of first-order reversal curves (FORC), Fig. 1(b) which will be explained next.

Ideally, distinct coercivities could be observed if an ensemble of different diameter MNWs were measured by standard hysteresis loops, where the moment (*M*) of the ensemble is measured as an applied field (*H*) is swept from +*H*_saturation_ to −*H*_saturation_ and back. However, instead of distinct coercivities, there is the shearing of the loop due to variations in the coercivities of the MNWs and/or due to dipole interactions between the MNWs. These two effects can be difficult to distinguish. However, FORC measurements were established [23]–[25] as a way to enable this distinction by measuring *M* as the field is stepped from a “reversal field” (*H*_*r*_ ) to +*H*_saturation_. Each line in Fig. 1(b) represents such a measurement for different values of *H*_*r*_. The FORC parameter is defined as

(1) $$\rho =-\frac{1}{2}\frac{\partial^{2}M_{\text{FORC}}\left(H,\;H_{r}\right)}{\partial H\;\partial H_{r}}.$$

This parameter is often plotted as a heatmap where the sample coercivities (*H*_*c*_) are represented in the *x* -direction and interaction fields (*H*_*u*_) are represented in the *y*-direction [see Fig. 1(c)]. Here, *H*_*c*_ (*H* − *H*_*r*_ )*/*2 and *H*_*u*_ (*H* + *H*_*r*_ )*/*2.

In rock magnetism, these FORC maps have been used to show the distributions of coercivities present in a sample (e.g., by different minerals) and the interaction fields that are experienced by each mineral [26]. In MNW arrays, however, many papers have disputed this interpretation, claiming that several features shown in Fig. 1(c) are artifacts of the measurement [27]–[29]. Even in rock magnetism, FORC heatmaps are sometimes only used as “fingerprints” to distinguish an unknown sample [28] within a library of samples. Unfortunately, even this decoding is limited by the measurement artifacts, e.g., due to smoothing, unless some quantitative analysis is performed to attempt the observations of coercivities [30].

Zamani *et al.* have shown that many features of the FORC map can be derived from projections ( *P*_*i*_ ) on the various axes (*i* ), where *i = H*_*r*_, *H*, *H*_*u*_, or *H*_*c*_ [29], see Fig. 1(c) (colored bell curves). More importantly, some of these projections only need the measurement of two points per curve rather than typical FORC with 50–100 measurements per curve, as mathematically shown by

(2) $$P_{\text{Hr}}=\int_{H_{r}}^{\infty }\rho \left(H,\;H_{r}\right)dH={\left.-\frac{1}{2}\frac{\partial M\left(H,\;H_{r}\right)}{\partial H_{r}}\right|}_{H=\infty }\;\;+{\left.\frac{1}{2}\frac{\partial M\left(H,\;H_{r}\right)}{\partial H_{r}}\right|}_{H=H_{r}}=0+{\left.\frac{1}{2}\frac{\partial M\left(H,\;H_{r}\right)}{\partial H_{r}}\right|}_{H=H_{r}}$$

(3) $$\begin{array}{l} P_{H}=\int_{-\infty }^{H}\rho \left(H,\;H_{r}\right)dH_{r}={\left.-\frac{1}{2}\frac{\partial M\left(H,\;H_{r}\right)}{\partial H}\right|}_{H_{r}=H}\\ \;+{\left.\frac{1}{2}\frac{\partial M\left(H,\;H_{r}\right)}{\partial H}\right|}_{H_{r}=-\infty }. \end{array}$$

As shown in (2), the projection of the FORC plot on the *H*_*r*_ -axis (*P*_Hr_) is simply the initial difference between FORC lines in Fig. 1(b). It is also interesting to note that this projection is equivalent to the irreversible switching field distribution (ISFD) because it is the amount of material that is not reversed as *H* is stepped back to the previous *H*_*r*_. Likewise, the projection on the *H* -axis ( *P*_*H*_ ) is equivalent to the initial slope of each FORC line (i.e., the reversible switching field (RSF) that is equal to the amount of material reversed as *H* is stepped back to the previous *H*_*r*_ ) minus the slope of lower branch of the hysteresis loop. Other features of the FORC data that could potentially be used in decoding mixtures of MNW barcodes are the backfield remanence (BMR) and the backfield remanence coercivity (BRC). As the names indicate, BMR is the moment remaining when *H =* 0 for each FORC line, and BRC is proportional to the difference between consecutive lines at *H* = 0 because it is the derivative of BMR (*∂ M*(*H, H*_*r*_)*/∂ H*_*r*_ *|*_*H*=0_).

**Fig. 2.**
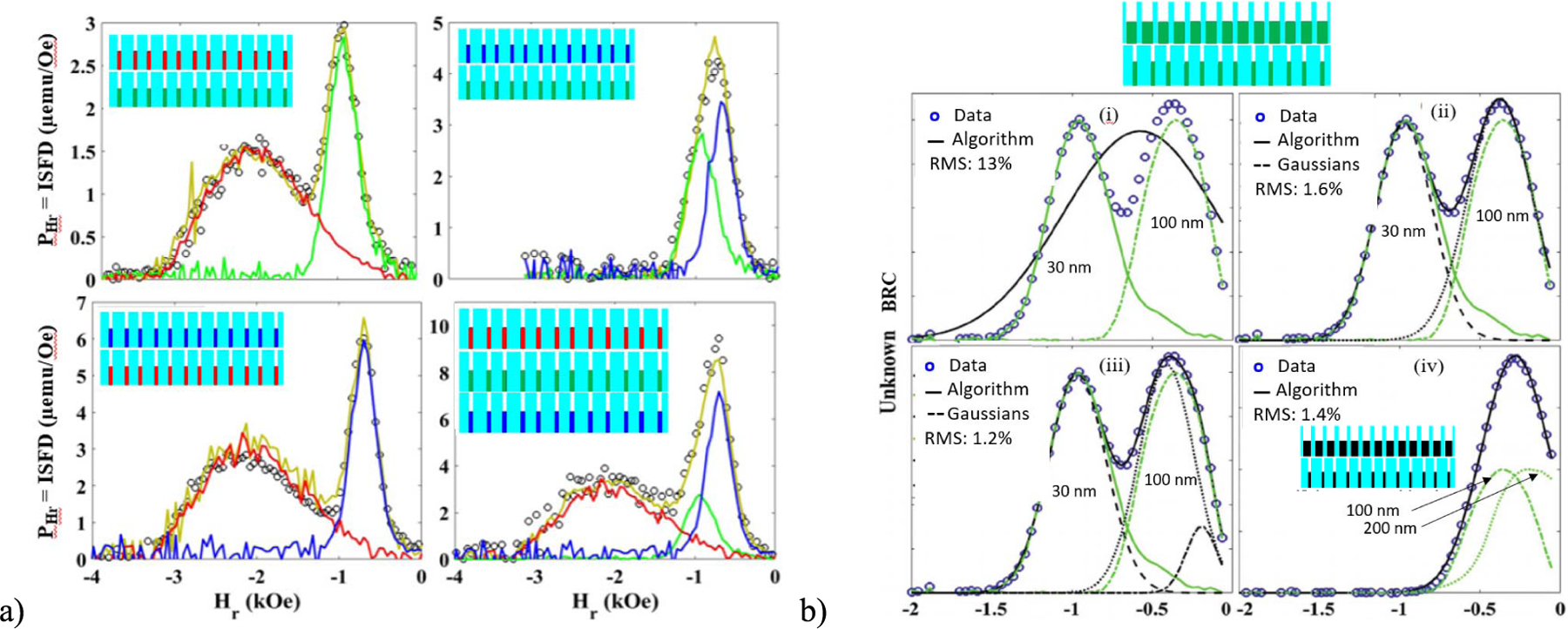
(a) Projections of FORC plots on the *H*_*r*_ -axis (equal to the ISFD) for combinations of two and three MNWs (Ni, Co, and FeCo) that were all 30 nm diameter in stacked arrays as shown [31]. Individual sample data are shown by solid color-coded lines, the combination data are shown as dots, and the identifying algorithm is a yellow line. (b) Examples (Ni) of backfield remnant coercivity (BRC) measurements for combinations of Ni and Co MNW (30, 50, 100, and 200 nm diameters each for eight single codes) where an algorithm (i)–(iii) correctly measured both *N =* 2 and the ID of codes when two codes were present, but (iv) failed another code due to overlapping BRC [21].

Of all the options above, ISFD and BRC have proven to be the most useful in decoding combinations of MNWs. For example, Fig. 2(a) shows *P*_Hr_(= ISFD) measured for combinations of two and three MNWs (Ni, Co, and FeCo) that were all 30 nm in diameter and 3 *μ*m long with large interwire distances (~6 *μ*m). In each case, a simple fit correctly identified the combination being measured (Exp data = Cal curve) [31]. In another study using Ni and Co MNWs with four different diameters each, Zamani developed an algorithm that correctly identified both how many codes were present and which MNWs made up those codes using BRC. Here, 3 *μ*m-long MNWs were used inside biocompatible polycarbonate with diameters/fill factors of 30 nm/0.5%, 50 nm/1.0%, 100 nm/1.7%, and 200 nm/12%. Successful identification was made for 26 of 28 combinations of two codes and 29 of 48 combinations of three codes. The failed coded combinations were double and triple combinations that included Ni (100 nm) with Ni (200 nm) and Co (30 nm) with Co (50 nm) because these BRC peaks overlapped as shown in Fig. 2(b)(iv) for Ni. These results were incorporated into a recent study of 3 *μ*m-long MNWs, where only 30 and 100 nm-diameter MNW codes were used for similar densities and FeCo MNWs were added to Ni and Co. In this study, all 15 double combinations were successfully decoded except for Co (100 nm) and FeCo (30 nm), and nine of the possible 20 triple combinations were successfully decoded.

**Fig. 3.**
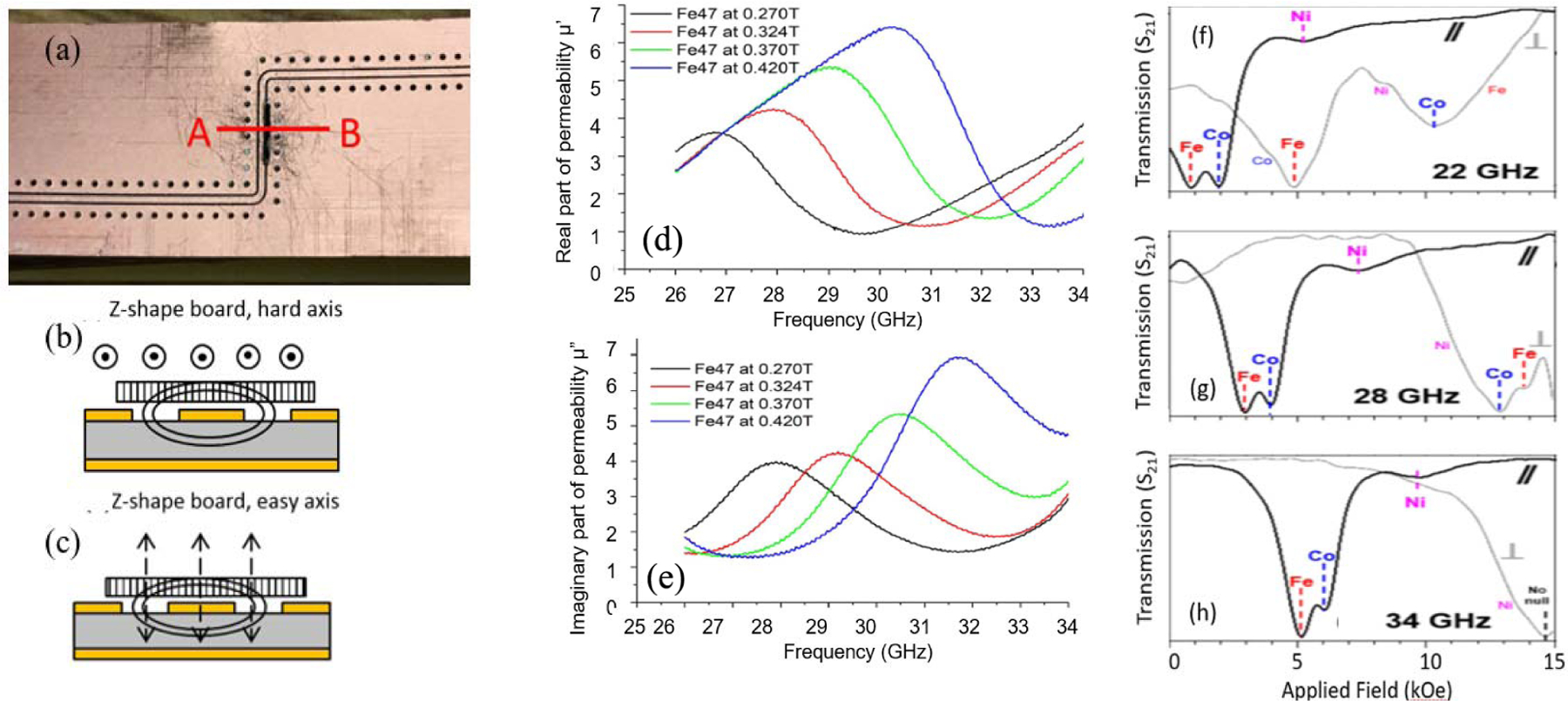
(a) Photograph of the coplanar waveguide (CPW) under the sample, which is the black rectangle with a cross-sectional line A and B. (b) and (c) Schematics of field geometries for two sample orientations once CPW is placed in a dc electromagnet. (d) and (e) Real and imaginary parts of the permeability of Fe MNW sample where a dc magnetic field was applied parallel to the MNWs and the frequency was swept from 24 to 34 GHz. (f) and (h) Ferromagnetic resonance identification (FMR-ID) of a barcode combination of three MNW samples at three frequencies with a dc field applied parallel and then perpendicular to the MNWs [34].

Another way to decode MNWs is ferromagnetic resonance (FMR) [33]. Fig. 3(a)–(c) shows the measurement geometry when MNWs are placed on a coplanar waveguide (CPW) and subsequently oriented inside an electromagnet to apply a dc field either parallel or perpendicular to the MNW axes (perpendicular or parallel to the plane of the sample, respectively). A vector network analyzer (VNA) sweeps frequency and the real and imaginary parts of the permeability are measured, as shown in Fig. 3(d) and (e) [34]. In addition, the moments of the MNWs also experience FMR at specific frequency–field pairs, as explained by the Kittel equation, which causes a loss of transmitted signal. Therefore, combinations of MNW samples can be decoded using high-frequency signal absorption as the decoding parameter. Fig. 3(e) and (f) shows the absorption at specific Kittel field–frequency pairs from a combination Fe, Co, and Ni MNWs inside nanoporous membranes with 18% fill factors. The MNW components present in the sample can be clearly identified using these absorptions, and several frequencies can be used to increase the robustness of the decoding as shown. These were 40 nm-diameter MNWs with lengths of ∼20 *μ*m.

## III. Biocomposites

**Fig. 4.**
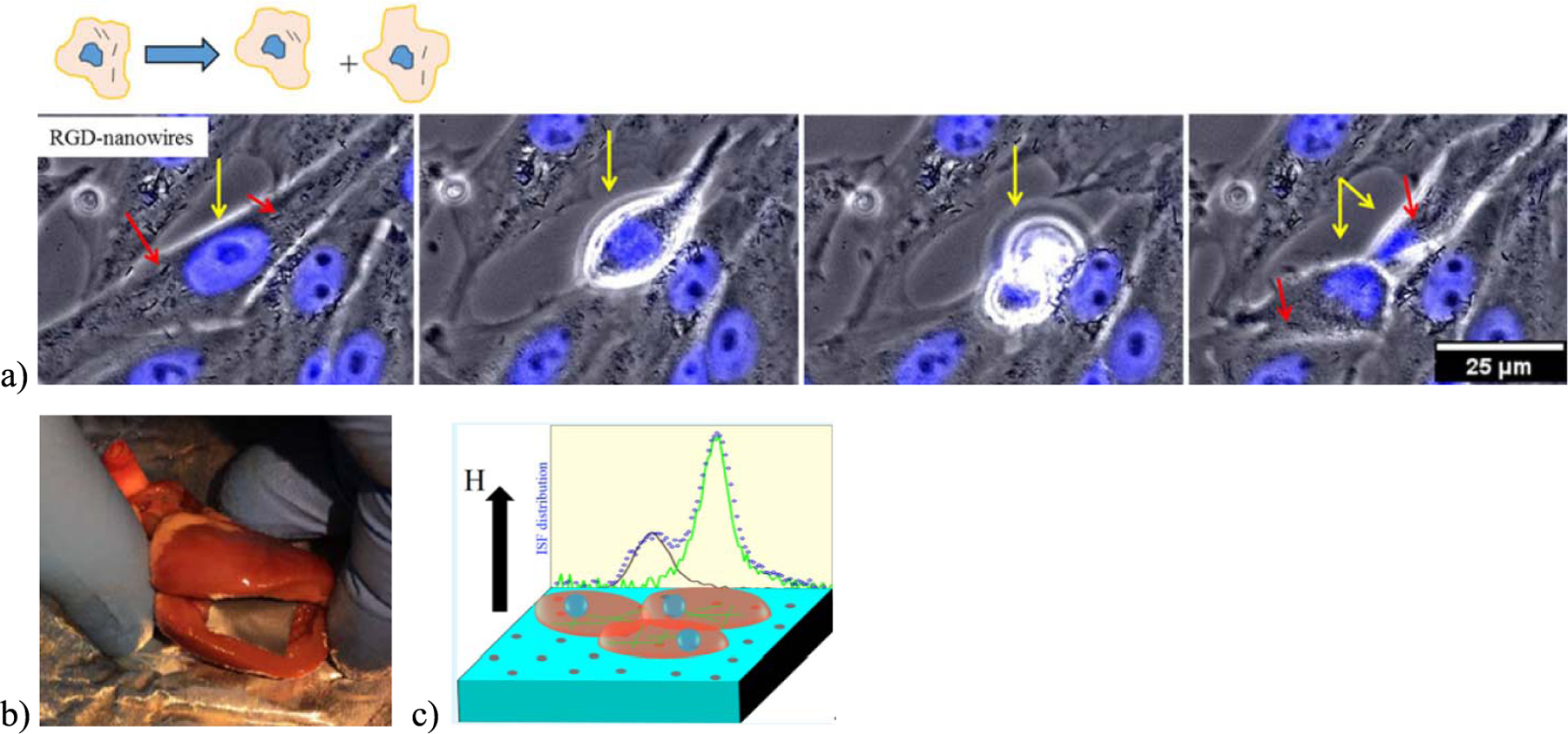
(a) Time-resolved strip showing an adherent osteosarcoma cell detaching from a Petri dish to divide and reattach (inset: schematic of the same to highlight the redistribution of MNWs inside daughter cells) [14]. (b) MNW-loaded biopolymer inside the heart of a model animal for measurement of BRC [12]. (c) Schematic showing MNW-loaded osteosarcoma cells on top of a biopolymer loaded with a different MNW code. The backdrop is the BRC data used to decode the cells from the biopolymer [36].

Starting in 2013, we have reported on the internalization of MNWs by cells [15]. Most types of cells were found to internalize MNWs if the cells and MNWs were incubated together at body temperature, for example, in phosphate-buffered saline (PBS). When labeled with the biomolecule peptide RGD, the MNWs exhibited self-dispersal by osteosarcoma cells [14]. Fig. 4(a) shows an adherent MNW-loaded osteosarcoma cell that disengages from the bottom of a Petri dish to divide, after which both daughter cells reattach with MNWs inside. These osteosarcoma cells were also observed to attach to the RGD-labeled MNWs and “walk away” with MNWs, thereby dispersing MNW clusters that had settled on top of the cells. It was an important observation that demonstrated how the magnetic attraction between MNWs was negligible partly due to the large separations between the poles of these highly anisotropic shapes and partly due to the RGD coating. Importantly, these MNWs were shown to have low cytotoxicity unless they are activated (e.g., via magnetic heating as discussed in the following).

As mentioned in Section I, MNW-embedded polymers could have applications in barcoded bandaids with read-out possible through external magnetic fields [12]. This means that internal patches can be detected without direct visualization or electrical contact. Although future studies will be needed to determine toxicity and lifetimes of such bandaids, the concept of decoding via external fields was verified by measuring a barcode inside a model organ [see Fig. 4(b)]. The decoding parameters of BRC and *P*_Hr_ were unchanged by the location of the barcode inside the heart of an animal model. In recent years, we have combined these bioapplications to show that MNW-loaded cells can be decoded from MNW-embedded biocomposites using the BRC and *P*_Hr_ parameters [see Fig. 4(c)].

## IV. Nanowarmers

**Fig. 5.**
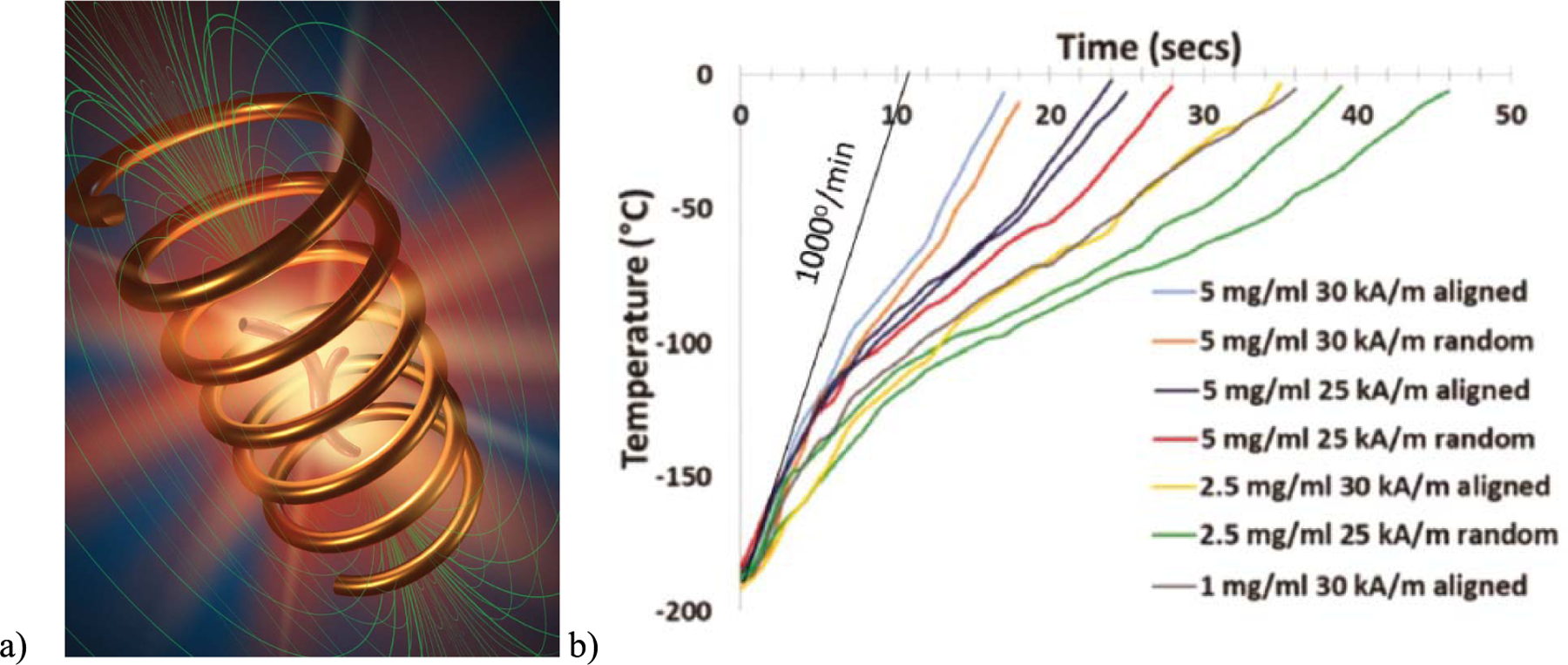
(a) Schematic of a blood vessel surrounded and filled with magnetic nanoparticles as they are warmed via magnetic reversal inside an alternating magnetic field [e.g., 500 Oe (20 kA/m) at 360 kHz]. (b) MNWs have proven to be very rapid nanowarmers in the cryopreservation agent MS55. Warming rates far exceed the necessary rate of 50 °C/min below which MS55 would crystallize on warming [18].

The last topic covered by this review is a recently discovered application of MNWs to rapidly warm cryopreserved specimens. Researchers have studied cancer therapy via hyperthermia for a decade or so [9], [37], [38]. In these studies, magnetic nanoparticles are labeled for specific attachment to cancer cells, and then, an ac magnetic field (e.g., 300 kHz) is applied via an external coil [see Fig. 5(a)]. The ac field causes rapid magnetization reversal and therefore induction loss, which manifests as heat. Sharma *et al.* [39] recently found that various magnetic nanoparticles can also be dispersed in cryopreservation agents (CPAs), which are biocompatible anti-freeze solutions that protect tissues and organs as they are vitrified in liquid nitrogen. To maintain viability as the specimens are warmed back to room (or body) temperature, the heating must be uniform to avoid cracking and rapid to avoid devitrification. This critical heating rate varies with the type of CPA, but a standard number is 50 °C/min for a CPA cocktail called MS55. Optimized spherical nanoparticles, such as superparamagnetic iron oxide nanoparticles (SPIONs), can reach the nanowarming rates of 130 °C/min with 500 Oe (20 kA/m) at 360 kHz and the concentrations of 10 mg/mL. MNWs have recently proven [18] to be excellent nanowarmers [see Fig. 5(b)], with the nanowarming rates of 400 °C/min with 1 mg MNW/mL CPA and 1000 °C/min with 5 mg/mL. Here, 200 nm-diameter, 8 *μ*m-long MNWs were isolated from their growth membranes and added to MS55 at the concentrations shown. The high anisotropy of MNWs works to their advantage if a small dc magnetic field is used to align the MNWs during vitrification of the specimen. It is interesting to note that MNWs faced resistance in this application due to their shape anisotropy. Many researchers envisioned MNWs causing “log jams” in capillaries, similar to what a lumberjack may face in a river. However, MNWs can be scaled to more closely represent pine needles in the Mississippi River, meaning that they will easily flow in capillaries as they are filled for cryopreservation and are rinsed out after rewarming.

## V. Conclusion

This review demonstrates the versatility of MNWs in bioapplications. Two methods for hands-off, contact-free read-out of bandaids and biolabels were presented. Namely, the magnetic signal of a sample is detected during a specific sequence of dc bias fields to enable mathematical projections of the sample’s FORC diagram using only a few measurement points compared to the standard FORC. In addition, high-frequency read-out of MNW biolabels was described. Finally, MNWs were shown to produce uniform, rapid warming of cryopreserved samples. It is important to realize that these features can be applied in concert with each other. For example, engineered MNWs inside biocomposites can be used as bandaids and barcodes. Also, cellular biolabels can be engineered to tag specific cells for read-out, followed by heating. Finally, free-floating MNWs stored in CPAs can be introduced to specimens undergoing vitrification, enabling subsequent read-out and magnetic nanowarming. The design parameters presented here are intended to help future medical physicists in the application of safe coding and cryopreservation.
